# Alkyl Pyridinol Compounds Exhibit Antimicrobial Effects against Gram-Positive Bacteria

**DOI:** 10.3390/antibiotics13090897

**Published:** 2024-09-20

**Authors:** Juan Canchola, Gracious Yoofi Boafo Donkor, Patrick Ofori Tawiah, Ayoola Fasawe, Emmanuel Ayim, Martin F. Engelke, Jan-Ulrik Dahl

**Affiliations:** 1 Department of Chemistry, Illinois State University, Normal, IL 61761, USA; 2 School of Biological Sciences, Microbiology, Illinois State University, Normal, IL 61761, USA; 3 School of Biological Sciences, Cell Physiology, Illinois State University, Normal, IL 61761, USA

**Keywords:** antimicrobial resistance, anaephenes, *Staphylococcus aureus*, membrane damage, biofilm formation

## Abstract

**Background/Objectives.** The rise of antibiotic-resistant pathogens represents a significant global challenge in infectious disease control, which is amplified by the decline in the discovery of novel antibiotics. *Staphylococcus aureus* continues to be a highly significant pathogen, causing infections in multiple organs and tissues in both healthcare institutions and community settings. The bacterium has become increasingly resistant to all available antibiotics. Consequently, there is an urgent need for novel small molecules that inhibit the growth or impair the survival of bacterial pathogens. Given their large structural and chemical diversity, as well as often unique mechanisms of action, natural products represent an excellent avenue for the discovery and development of novel antimicrobial treatments. Anaephene A and B are two such naturally occurring compounds with significant antimicrobial activity against Gram-positive bacteria. Here, we report the rapid syntheses and biological characterization of five novel anaephene derivatives, which display low cytotoxicity against mammalian cells but potent antibacterial activity against various *S. aureus* strains, including methicillin-resistant *S. aureus* (MRSA) and the multi-drug-resistant community-acquired strain USA300LAC. **Methods.** A Sonogashira cross-coupling reaction served as the key step for the synthesis of the alkyl pyridinol products. **Results/Conclusions.** Using the compound JC-01-074, which displays bactericidal activity already at low concentrations (MIC: 16 μg/mL), we provide evidence that alkyl pyridinols target actively growing and biofilm-forming cells and show that these compounds cause disruption and deformation of the staphylococcal membrane, indicating a membrane-associated mechanism of action.

## 1. Introduction

The rise of antibiotic-resistant pathogens represents a significant global challenge in infectious disease control, which is amplified by the decline in the discovery of novel antibiotics. Antibiotic-resistant infections, which affect more than 2.8 million people and cause over 35,000 deaths each year in the US alone [1], have emerged in all antibiotic classes and are projected to increase worldwide to 10 million deaths annually by 2050 [2]. They are particularly challenging for at-risk populations such as immunocompromised patients [3]. *Enterococcus faecium*, *Staphylococcus aureus*, *Klebsiella pneumoniae*, *Acinetobacter baumannii*, *Pseudomonas aeruginosa*, *Enterobacter species*, and *Escherichia coli* (commonly described as ESKAPEE pathogens) represent the leading cause of nosocomial infections and are considered most concerning due to their reported multi-drug resistance [4]. *S. aureus* is a Gram-positive commensal that readily colonizes the human skin and nasal cavities without causing any harm to the host [5,6]. However, *S. aureus* also represents one of the most serious human bacterial pathogens, causing a variety of nosocomial and community-acquired infections, ranging from mild skin infections and food poisoning to life-threatening septicemia, toxic shock syndrome, and endocarditis [6,7]. *S. aureus* is capable of readily developing resistance mechanisms against antibiotic treatments as well as evading clearance by immune cells [8,9], making the pathogen incredibly challenging to eradicate. In fact, it is estimated that 90–99% of *S. aureus* isolates are resistant to the β-lactam antibiotic penicillin, and a significant number of nosocomial infections are caused by methicillin-resistant *S. aureus* strains (MRSA) [10,11]. However, the antibiotic resistance of MRSA strains is not only limited to methicillin but extends to aminoglycoside, fluoroquinolone, and macrolide antibiotics [11,12]. While significant progress has been made in the field of antimicrobial chemotherapy, such as the use of the membrane-disrupting lipopeptide antibiotic daptomycin [13], the continued prevalence of *S. aureus* infections prompts the need for additional alternative therapies [5].

The multi-functional plasma membrane is a prominent structure in the life of a bacterium, forming an essential boundary to the extracellular space, regulating membrane protein function, and controlling the entry and exit of various molecules, including antibiotics and other antimicrobial agents. Apart from its protective function, it also serves as a site for respiration, active transport of materials, ATP production, and signal transduction [14,15,16]. More recently, it has been shown that *S. aureus* can incorporate exogenous fatty acids into its membrane to regulate fluidity, enhancing its growth and adaptation under temperature stress [17]. The membrane is the first site of attack for antimicrobial peptides and other components of the innate immune system [18]. As such, antimicrobials with membrane-disrupting mechanisms of action may serve as optimal avenues for treating *S. aureus* infections.

Many of the antibiotics currently in use, including β-lactams, aminoglycosides, tetracyclines, macrolides, and glycopeptides, have originally been isolated from nature where microorganisms produce them to fight potential competitors [19]. These natural products have a vast range of structural diversity and provide scientists with new molecular scaffolds for further development into more potent therapeutics, hopefully resulting in more efficient treatments of infections [20,21]. Considering that the purpose of natural products is to interact with biological systems and given their vast chemo diversity and variety of unique mechanisms of action available, natural products are an excellent avenue for antibiotic discovery [22]. The development of novel drug molecules from natural products is often restricted by issues in the isolation, supply, and characterization processes, which, however, can be overcome by synthesizing natural products from scratch [23,24]. Additionally, the derivation of natural products offers an opportunity to increase the potency against their microbial targets, reducing off-target effects on symbiotic microbes and reducing toxicity to the patient.

Recently, the syntheses and structure-activity studies of anaephenes A and B, as well as eighteen derivatives, have been reported [25,26]. These compounds are part of the alkylphenol family, demonstrating a wide variety of biological activities, including selective inhibition of the concanavalin A-induced interleukin-2 production in Jurkat cells [27]. Among these newly synthesized compounds were alkylphenol analogs, particularly compound **18**, that exhibited potent antimicrobial activities against MRSA while also displaying very little to no cytotoxicity towards red blood cells [26]. However, their antimicrobial mechanism of action is still unknown. Therefore, in this study, we aimed to design, synthesize, and biologically assess optimized anaephene analogs with improved drug-like properties. Due to their high lipophilicity, aliphatic structure, and proposed specificity for Gram-positive bacteria such as *S. aureus*/MRSA, we hypothesized that the antimicrobial mechanism of action of anaephene compounds is based on its integration into the bacterial membrane, potentially leading to membrane disruption and ultimately causing cell death. Here, we report particularly one new compound, named JC-01-074, which displays bactericidal activity at low concentrations (MIC: 16 μg/mL), targets actively growing and biofilm-forming cells, and displays relatively low toxicity to mammalian 3T3 cells (IC_50_: 24.6 µg/mL). Additional studies with fluorescent dyes provide evidence that this anaephene derivate disrupts and deforms the staphylococcal membrane, suggesting that its mechanism of action is membrane-associated.

## 2. Results

### 2.1. Synthesis of Alkyl Pyridinol Analogs with Variations in the Position of the Nitrogen Atom

**Figure 1:**
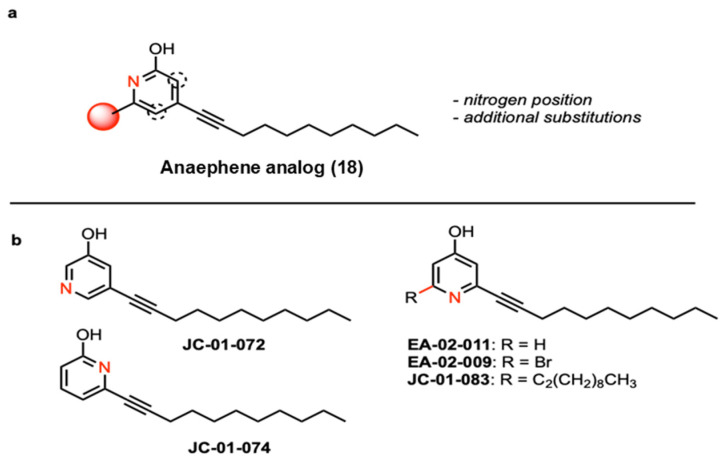
Structural formulas of the alkyl pyridinol compounds. (**a**) The previously published anaephene compound **18** was modified, resulting in compounds with the nitrogen atom at different positions (dotted circle) and/or additional substitutions (red sphere). (**b**) Analogous compounds were generated with the nitrogen atom oriented in meta (JC-01-072) and ortho (JC-01-074), respectively, and para (EA-02-011) to the alcohol. Additional analogs JC-01-83 (di-alkyl chain) and brominated JC-02-009 were generated to examine the effects of an additional alkyl chain and a large electronegative atom.

A recent study reported that compound **18** (Figure 1a), a 2-hydroxypyridine, displays significant antimicrobial activity while also being characterized by remarkably lower cytotoxicity towards red blood cells compared to anaephene B [26]. Based on these findings, we designed and conducted the syntheses of five novel alkyl pyridinol compounds, which differ in the position of the nitrogen atom in the phenol ring, allowing us to assess the significance of the nitrogen position as well as lipophilicity for the antimicrobial activity of each compound (Figure 1 and Figure S1). As reported before, the general procedure of the synthesis involved a Sonagashira cross-coupling reaction [25,26]. However, instead of an alkyl phenol head group, we utilized pyridine to generate the alkyl pyridine group of the five compounds, as this increases compound hydrophilicity and improves the hydrogen-bonding capabilities—two important drug properties [28]. The use of pyridines is also favored for drug discovery due to their ability to act as bioisosteres of amines, amides, and nitrogen-containing heterocycles [29]. By utilizing different bromo- or iodo-substituted pyridines as educts, we obtained the four compounds JC-01-72, JC-01-74, JC-01-083, and EA-02-009 as clear-yellow oils (Supplementary Figure S1). We further removed the bromine from EA-02-009 by hydrogenation with n-BuLi, resulting in the brownish-colored compound EA-02-011. In compound JC-01-072, the nitrogen atom is located in the meta position from the hydroxyl group, whereas it is found in the ortho position in JC-01-074 and para position in EA-02-011 (Figure 1 and Figure S1). Two analogs allow us to examine the effects of adding an additional alkyl chain (JC-01-083) and a large electronegative halogen such as bromine (EA-02-009). All synthesized products were monitored by TLC analysis (silica gel 60 F254, 250 mm layer thickness) and visualized with a 254 nm UV light. The compounds were purified by flash column chromatography performed with silica gel 60 (230–400 mesh) and their structures confirmed by 1H and 13C NMR spectroscopy as well as high-resolution mass spectrometry (described in the Supplementary Material and Methods, Supplementary Figures S4–S13).

### 2.2. Anaephene Derivates Exhibit Antimicrobial Activity Selectively against Gram-Positive Bacteria

In previous studies, anaephene compounds have been reported to exhibit potent antimicrobial activities against Gram-positive bacteria such as *S. aureus* [26]. To assess the impact of the nitrogen position in our newly synthesized anaephene derivates, we analyzed their antimicrobial activities against various *S. aureus* strains, including ATCC 25,923 and the two methicillin-resistant *S. aureus* (MRSA) strains ATCC 33,591 and ATCC BAA-44. We also tested these compounds against a strain of the Gram-negative opportunistic pathogen *Pseudomonas aeruginosa*, ATCC 27853, as previous anaephene analogs were shown to be highly specific towards Gram-positive bacteria and completely ineffective against Gram-negatives [26]. We determined the minimum inhibitory concentrations (MIC) of each of our compounds against the different bacterial strains by broth microdilution following the CLSI guidelines [30]. As expected, all five compounds tested were completely ineffective against the Gram-negative *P. aeruginosa* strain ATCC 27,853 (Table 1). EA-02-009 was the most potent compound against *S. aureus*/MRSA, with MIC values between 0.5–1 μg/mL, depending on the *S. aureus* strain tested. Slightly less potent were compounds JC-01-072 and JC-01-074, with MIC values of 4 and 16 μg/mL, respectively. JC-01-083 was inactive against any of the *S. aureus*/MRSA strains tested (Table 1).

### 2.3. JC-01-074 Possesses Bactericidal Activity

Considering the potency of the alkyl pyridinol analogs at limiting Gram-positive growth, we explored their activities against the multi-drug-resistant community-acquired *S. aureus* strain USA300LAC. Following the CLSI broth microdilution guidelines [30], we determined the MIC values for JC-01-072, JC-01-074, and EA-02-009, respectively, which were with 8 µg/mL, 16 µg/mL, and 32 µg/mL significantly higher for USA300LAC compared to the other three *S. aureus*/MRSA strains tested (Table 1). To determine whether one of the three compounds exhibits bactericidal effects, we determined their minimal bactericidal concentrations (MBC). Both JC-01-072 and EA-02-009 showed high MBC values (Table 1), indicating that these compounds mainly elicit bacteriostatic effects. JC-01-074, in contrast, is bactericidal and completely eradicates USA300LAC at its MIC of 16 μg/mL (Table 1).

**Figure 2:**
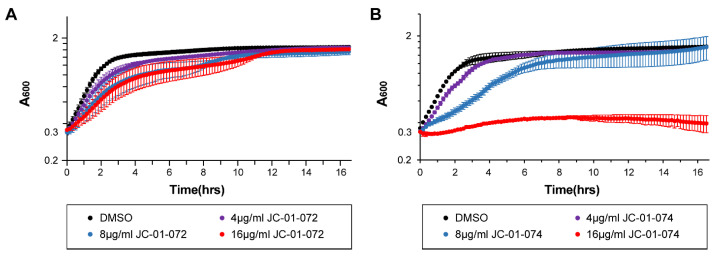
Impact of alkyl pyridinol exposure on exponentially growing *S. aureus*. An overnight culture of the *S. aureus* strain USA300LAC was diluted into fresh TSB and grown to mid-exponential phase (OD_600nm_ = 0.2–0.3) prior to treatment with 4–16 µg/mL JC-01-072 (**A**) and JC-01-074 (**B**). Optical densities were measured at 600 nm at 10 min intervals for 16–18 h (*n* = 3, ±S.D.).

During the exponential phase, bacterial cells exhibit increased sensitivity to antimicrobials, a phenomenon that is less pronounced in stationary phase cells due to the upregulation of additional stress defense systems and a reduced growth rate when nutrients become limited [31,32]. Many antimicrobials are designed to target actively dividing bacterial cells and, therefore, exhibit higher potencies against exponentially growing cells [33]. Considering the divergent antimicrobial effects of our new compounds, we proceeded to assess the impact of JC-01-072 and JC-01-074 on the growth of exponentially growing USA300LAC cells. We performed growth-curve-based assays of the USA300LAC cultures that were either left untreated or treated with 4–16 μg/mL of the alkyl pyridinol compound once they reached the mid-log phase (OD_600nm_~0.2–0.3). Exposure to JC-01-072 had little to no effects on the growth of the mid-log USA300LAC cells at any of the concentrations tested (Figure 2A). On the other hand, we found that 0.5× MIC of JC-01-074 delayed the growth of USA300LAC by approximately 6–8 h. Treatment with 1× MIC resulted in a total growth arrest of USA300LAC (Figure 2B), suggesting that actively growing cells are quite sensitive to low concentrations of this compound.

### 2.4. JC-01-074 Inhibits Biofilm Formation but Does Not Eradicate Established Biofilm

**Figure 3:**
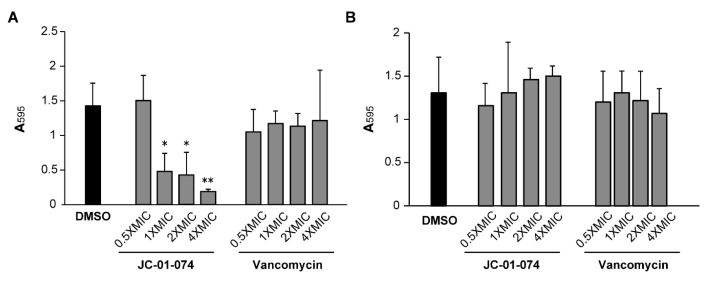
JC-01-074 inhibits *S. aureus* biofilm formation but does not eradicate established biofilms. Overnight cultures of *S. aureus* strain USA300LAC were diluted into fresh TSB to an OD_600nm_ = 0.001 and incubated aerobically at 37 °C until the mid-log phase. (**A**) To detect effects on biofilm formation, cultures were diluted to an OD_600nm_ = 0.0001 in the presence and absence of JC-01-074 and vancomycin at concentrations of 0.5–4× MIC, respectively. (**B**) To detect effects on biofilm eradication, cultures were diluted to an OD_600nm_ = 0.0001 and incubated under static conditions for 24 h, after which planktonic cells were removed, and the preformed biofilms were washed twice with PBS. Biofilms were incubated with fresh TSB supplemented with JC-01-074 and vancomycin at 0.5–4× MIC for 24 h, respectively. Biofilm mass was quantified after 20 h via crystal violet staining. One-way ANOVA, Dunnett’s multiple-comparison test (* *p* < 0.05, ** *p* < 0.01); (*n* = 3, ±S.D.).

Chronic persistent infections are frequently caused by pathogenic bacteria living in biofilm communities [34]. The establishment of a bacterial biofilm usually begins with pathogen attachment to biotic and abiotic surfaces [35], and *S. aureus* is notorious for its ability to form biofilms on surfaces, such as implants and catheters, which presents a unique treatment challenge [36,37]. Studies of membrane-targeting antimicrobials have revealed their potential for curtailing biofilm formation [38,39]. Given that JC-01-074 is the only compound exhibiting low MIC and MBC values (Table 1), we explored its potential to inhibit the formation of new biofilm communities and to disperse already established biofilms in comparison to vancomycin [40,41,42]. Our data indicate that treatment with JC-01-074 reduced the biofilm formation of the *S. aureus* strain USA300LAC by ~60–70% in a dose-dependent manner (Figure 3). In contrast, the vancomycin treatment failed to significantly inhibit biofilm formation, even at 4× MIC. However, neither JC-01-074 nor vancomycin successfully reduced established biofilms at the concentrations tested, ruling out potential biofilm eradication properties when used at or below 4× MIC. In summary, our data suggest that JC-01-074 prevents USA300LAC from forming biofilm but has no effect once biofilms have been established.

### 2.5. The New Alkyl Pyridinol Derivates Showed Slight Cytotoxicity in Mammalian Cell Lines

To gauge the potential for toxicity of the new alkyl pyridinol derivates, we tested their cytotoxicity by examining the metabolic activity of 3T3 mouse embryonic fibroblast cells using the tetrazolium dye 3-(4,5-dimethylthiazol-2-yl)-2,5-diphenyltetrazolium bromide (MTT), as has been done before [43]. The dye is reduced by NAD(P)H-dependent cellular oxidoreductases, resulting in the formation of the insoluble formazan with its purple color, which can be quantified [44]. 3T3 cells were treated with different concentrations of the alkyl pyridinol compounds for 24 h, and the absorbance of the MTT-treated cell culture supernatant was measured at 540 nm. Absorbances were plotted against concentrations of the compound added (Supplementary Figure S2), and IC50, the concentration of a drug required for 50% metabolic inhibition, was calculated for each compound. Our data revealed that JC-01-072 shows the highest IC50 (37.7 ± 8.3 µg/mL), suggesting it to be the least toxic compound for 3T3 cells (Table 2 and Supplementary Figure S2). Treatment with EA-02-009 (34.5 ± 26.7 µg/mL), JC-01-074 (25.6 ± 2.3 µg/mL), and JC-01-083 (31 ± 9.5 µg/mL) resulted in slightly lower IC50 concentrations, indicating a higher cytotoxicity of each compound. However, the concentrations at which these compounds compromise 3T3 mouse embryonic fibroblast cells are well above their reported MICs (Table 2). EA-02-011 is the alkyl pyridine derivate with the lowest IC50 (10.7 ± 2 µg/mL), indicating cytotoxicity already at low concentrations. Overall, our data suggest that there might be a small therapeutic window for some of the newly synthesized compounds.

### 2.6. JC-01-074 Causes Significant Membrane Damage

**Figure 4:**
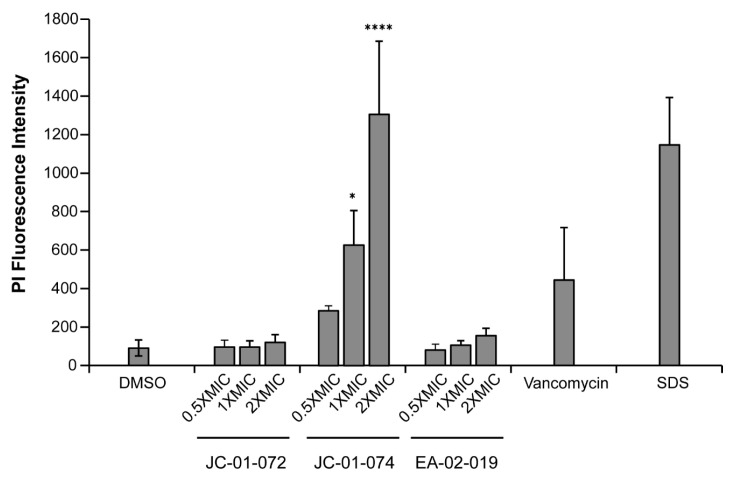
JC-01-074 causes significant membrane damage in *S. aureus*. Membrane damage was evaluated via propidium iodide (PI) staining. Exponentially growing USA300LAC cells were treated with the indicated alkyl pyridine compounds at 0.5–2× MIC for 1 h. Treatment of USA300LAC with 2.5% DMSO, 4 µg/mL vancomycin, and 0.25% SDS were included as negative and positive controls, respectively. Cells were collected, washed, resuspended in PBS, and stained with 1 µM PI. PI fluorescence was measured (exc./em. wavelengths: 535/615 nm) and normalized to OD_600nm_. One-way ANOVA, Dunnett’s multiple-comparison test (* *p* < 0.05, **** *p* < 0.0001); (*n* = 3, ±S.D.).

The alkyl pyridinol derivates synthesized in this study are characterized by high lipophilicity. To determine whether and to what extent these compounds incorporate into and disrupt the phospholipid bilayer of the *S. aureus* cell envelope, we performed a propidium iodide (PI) uptake assay [45]. Given the membrane-impermeable nature of PI, the fluorophore can be used to assess membrane disruption in cells by measuring its non-specific interaction with DNA, which leads to enhanced fluorescence [46]. We found that 1 h treatments with JC-01-074 at its MIC (i.e., 16 µg/mL) resulted in about five-fold higher PI fluorescence compared to the DMSO-treated control, which was comparable to treatments with known membrane-disrupting agents, such as vancomycin and sodium dodecyl sulfate (SDS), respectively (Figure 4). The extent of membrane damage caused by JC-01-074 occurred in a dose-dependent manner. Conversely, JC-01-072 and EA-02-009 exerted minimal changes in the PI intensity, even at 2× MIC after 1 h of exposure (Figure 4). Overall, our data suggest that JC-01-074 likely exhibits its high bactericidal activity by causing extensive damage to the *S. aureus* membrane.

### 2.7. JC-01-074 Rapidly Induces Cell Membrane Deformations

**Figure 5:**
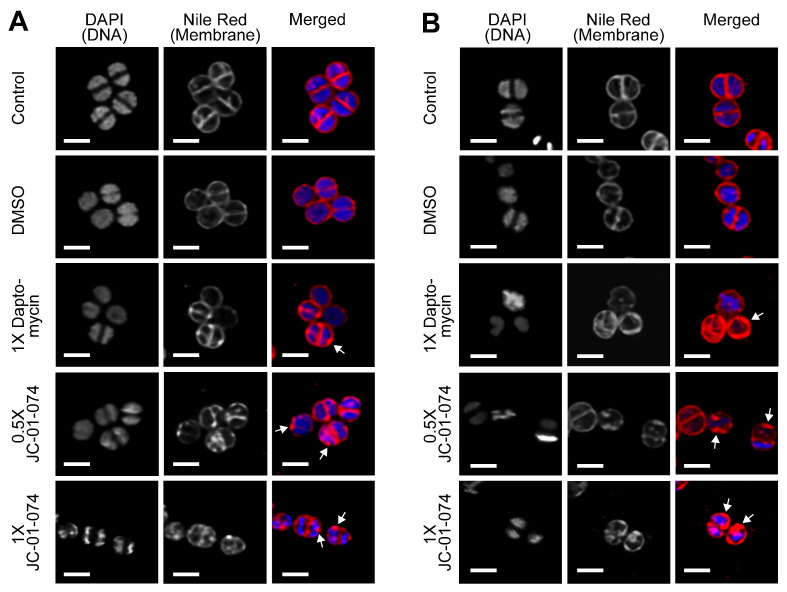
JC-01-074 treatment rapidly induces deformations in the *S. aureus* plasma membrane as indicated by the white arrows. USA300LAC cells were grown aerobically to mild-log phase in TSB before they were either left untreated or treated with 2.5% DMSO, 4 µg/mL daptomycin (1× MIC), 8 µg/mL JC-01-074 (0.5× MIC), or 16 µg/mL JC-01-074 (1× MIC) for 15 min (**A**) and 60 min (**B**), respectively. Cells were collected at the indicated time points and stained with DAPI (5 µg/mL) and Nile Red (5 µg/mL) for 10 min, following which cells were imaged on 1% agarose pads. Images are representatives of three biological replicates. Scale bar = 1.25 µm.

Given the high lipophilic nature and membrane-compromising effects of JC-01-074, we wondered about the possibility that the alkyl chain of JC-01-074 could be incorporated into the phospholipid bilayer, thereby destabilizing the uniform packing of the membrane phospholipids and potentially resulting in “bleb formation”, as this had been reported before [47]. To test this idea, we cultivated the *S. aureus* strain USA300LAC to the mid-log phase and treated the cells with the indicated concentrations of JC-01-074 and daptomycin (as a positive control), respectively. Samples were collected at 15 and 60 mins of treatment, washed twice, and resuspended in fresh PBS. Cells were then stained with 4′,6-diamidino-2-phenylindole (DAPI) and Nile Red for 10 min, respectively. DAPI is a nucleic acid stain that binds to DNA, while Nile Red is commonly used as an inner membrane stain to quantify lipids. The cells were then mounted onto a glass slide with 1% agarose and imaged at 63× magnification via inverted confocal laser scanning microscopy. We started to observe bleb formation in the *S. aureus* plasma membrane as early as 15 min after the addition of JC-01-074 and at concentrations as low as 8 µg/mL (i.e., 0.5× MIC) (Figure 5A). Treatment with the vehicle control DMSO did not show any obvious changes on the cell surfaces. To rule out the possibility that these blebs were transiently formed, we further imaged the cells exposed to JC-01-074 for 60 min. Both treatments with JC-01-074 at 0.5× and 1× MIC resulted in the formation of aberrant membrane blebs and disrupted the overall architecture of the cell membrane (Figure 5B). The smaller blebs formed may have extended over the incubation time, resulting in their fusion into large blebs. Overall, our data suggest that, like doxycycline, JC-01-074 also exhibits its high bactericidal activity by forming blebs in the *S. aureus* membrane, which is in good agreement with the proposed chemical nature of this compound.

### 2.8. JC-01-074 Has No Significant Synergistic Effects with Clinically Relevant Antibiotics

**Figure 6:**
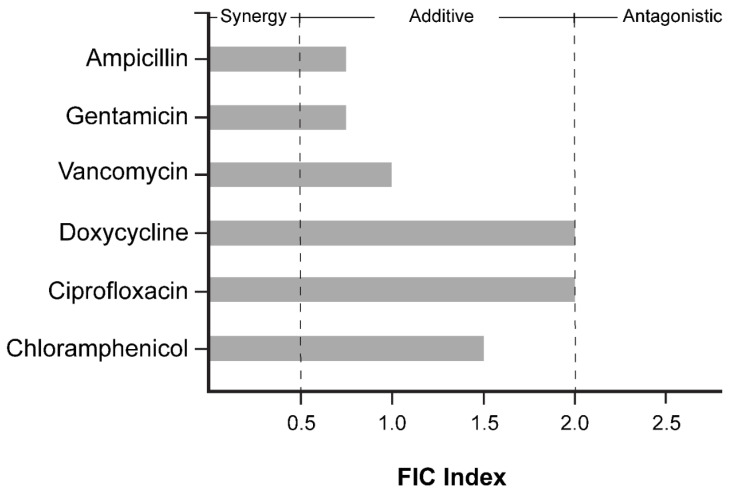
FIC index of JC-01-074, tested in combination with ampicillin, gentamicin, ciprofloxacin, doxycycline, vancomycin, and chloramphenicol. FICs were calculated as the ratio of the MIC of the antimicrobial in combination with the MIC of the antimicrobial alone. Interactions were categorized as synergistic (<0.5), additive or no interaction (0.5–2), and antagonistic (>2). FICs were calculated from three biological replicates.

Membrane disruption in both Gram-positive and Gram-negative bacteria has been demonstrated to increase the potency of certain conventional antibiotics [38,39]. Therefore, we tested whether the membrane-disrupting effects of JC-01-074 could increase the sensitivity of the *S. aureus* strain USA300LAC to antibiotics of different classes; for instance, by facilitating their uptake into the cytoplasm. Six antibiotics of the groups of fluoroquinolones (i.e., ciprofloxacin), aminoglycosides (i.e., gentamycin), glycopeptides (i.e., vancomycin), tetracyclines (i.e., doxycycline), β-lactams (i.e., ampicillin), and chloramphenicol were tested at the indicated concentrations with and without increasing concentrations of JC-01-074 in a checkerboard assay, following the established protocols [48]. Antibiotic concentrations were prepared by the microbroth dilution in MHB based on their MIC values (Table 1) to obtain the final concentrations as outlined in the 96-well plate scheme for each antibiotic tested (Supplementary Figure S3). Overnight USA300LAC cultures were diluted to 5 × 10^5^ CFU/mL in MHB, and the growth was recorded at 37 °C and 200 rpm for 20 h in the presence and absence of the indicated antibiotic. Fractional inhibitory concentration (FIC) calculations were computed using the lowest concentration of both antimicrobials that completely inhibited bacterial growth. The sum of the FIC values indicates that no antibiotic tested interacted synergistically with JC-01-074 (Figure 6 and Figure S3). Ampicillin, gentamicin, and vancomycin had additive effects on the killing efficiency of JC-01-074, while other antibiotics, such as doxycycline and ciprofloxacin, showed mild antagonistic effects with JC-01-074.

## 3. Discussion

As previously reported, the anaephene derivative compound **18** exhibits significant antimicrobial activity, which was comparable to that of its closely related natural product, anaephene B [26]. Importantly, compound **18**, a 2-hydroxypyridine analog, was significantly less cytotoxic to mammalian cells as it showed an ~80% reduction in red blood cell lysis compared to equivalent concentrations of anaephene B [25]. The reduced cytotoxicity of compound **18** was attributed to the addition of a nitrogen atom in the pyridine headgroup, which potentially increases the hydrophilicity and polarity of the molecule despite its otherwise lipophilic nature due to its long-alkyl chain (Figure 1). Lipophilicity is an important property to consider in drug development, given its profound impact on drug absorption and the metabolism of drugs. Moreover, increased lipophilicity may promote off-target binding and potentially result in increased toxicity [49]. Thus, the introduction of heteroatoms into the ring increases polarity and hydrophilicity in molecules and thus represents a possibility to limit toxicity. Heterocyclic compounds are also thought to be more versatile in drug development due to their increased hydrogen-bonding capabilities, which in turn may lead to improved pharmacological, pharmacokinetic, toxicological, and physicochemical properties of the drug [50].

The goal of this study was to further improve the antimicrobial properties of compound **18**. To characterize whether and to what extent the position of the nitrogen atom in the phenol ring plays a role in the antimicrobial activity, we synthesized alkyl pyridinol analogs with nitrogen atoms positioned at various locations in the phenol ring, resulting in compounds JC-01-072 (meta), JC-01-074 (ortho), and EA-02-011 (para). In addition, JC-01-083 was di-substituted with two long-alkyl chains to investigate the effects of increased lipophilicity, while EA-02-009 carries a large electronegative bromine at the meta position. By studying the effects on antimicrobial activity that these small structural changes may elicit, we can start shedding light on the mechanism of action of alkyl pyridinol, further improve biological activities, and potentially identify a lead compound. An assessment of their biological activity was first conducted by determining the MICs in a variety of different *S. aureus* strains, including methicillin-sensitive and MRSA strains, as well as a Gram-negative *P. aeruginosa* strain. EA-02-009, which carries the bromine, was most efficient in inhibiting the growth of different *S. aureus*/MRSA strains, with MIC values ranging from 0.5–1 μg/mL. Surprisingly, EA-002-009 was much less potent against the MRSA strain USA300LAC, which required 32 µg/mL of the compound to completely inhibit growth. We predict that the large size and electronegativity of the bromine atom could increase the reactivity of the compound and/or modulate potential interactions. Bromine is also a good leaving group and may, therefore, increase the nucleophilic character of the compound [51]. While JC-01-072 is less toxic than EA-002-009 with MICs between 4–8 µg/mL, both compounds act bacteriostatically, as no significant killing was observed, even at concentrations of 128 μg/mL. JC-01-074 and EA-02-011 showed lower toxicity towards the *S. aureus*/MRSA strains tested, with MIC values of 16 and 32 μg/mL, respectively, indicating that the position of the nitrogen in the phenol ring of the pyridinol has some effects on the antimicrobial activity of the compound. The compound carrying the nitrogen in meta orientation (i.e., JC-01-072) yielded the best antimicrobial activity compared to JC-01-074 (ortho) and EA-02-011 (para). The meta position may improve the stability of the compound, which is supported by previous studies of analogs with the meta-orientated hydroxy group and alkyl chain that improved antimicrobial properties [52]. We rationalize the variance in biological activities between JC-01-072, JC-01-074, and EA-02-011 with the principles of aromatic resonance between electron withdrawing and donating groups. Pyridine resonance induces nucleophilic susceptibility, and electron-donating groups, such as secondary alcohols, direct electrophilic substitution [53]. Switching the nitrogen position changes these directing properties and, therefore, may play a role in the antimicrobial properties of the molecule. JC-01-083 was completely inactive against *S. aureus*/MRSA, likely due to its high lipophilic character. All the analogs were completely inactive against the Gram-negative pathogen *P. aeruginosa*, suggesting that the compounds act via the mechanism of action that only effectively targets Gram-positive bacteria.

Notably, synthesis of the alkyl pyridinol with the nitrogen atom in the ortho position (i.e., JC-01-074) rendered the compound bactericidal, and concentrations as low as the MIC were sufficient to completely eradicate the *S. aureus* strains tested. Given its bactericidal mode of action and relatively low MIC/MBC, the applicability of JC-01-074 was investigated in more detail. Most bacterial pathogens, including *S. aureus*/MRSA, are known to attach to either biotic or abiotic surfaces to form biofilm communities [54,55]. This lifestyle switch provides many survival benefits, including protection against immune cell attack, shared metabolism, and up to 1000-fold increased resistance to antibiotic treatment [56]. While JC-01-074 had no significant effects on the already established biofilms, it was found to be very potent in inhibiting their formation.

All five compounds displayed moderate cytotoxic activity against 3T3 mice fibroblasts. Generally, compounds are considered toxic when the metabolic activity of the eukaryotic cells is compromised at or below 30 µg/mL [57]. All compounds tested showed moderate levels of cytotoxicity, with IC50 values ranging from 25–38 μg/mL. Toxicity levels are an important factor in consideration of potential drug candidates; however, what is more important is the therapeutic index, which reflects the ratio between the therapeutic concentration range of a drug and the lowest concentration at which toxicity is observed [58]. In drug deployment, therapeutic indices (Tis) of ~10 or a difference of at least 10-fold are typically desired. However, drugs with lower TIs are often still considered safe and effective if their benefits outweigh the risks [58]. Because we only assessed compound toxicities in vitro and in non-clinical settings, extrapolating the therapeutic indices would not be appropriate. Taking our data from the MIC and cytotoxicity studies into consideration, we can cautiously speculate which compounds likely yield larger safety windows and/or acceptable therapeutic indices. Compounds JC-01-072, JC-01-074, and EA-02-009 displayed the highest IC50 concentrations between 25–38 µg/mL. Meanwhile, the MIC values of these three compounds were between 0.5 and 16 μg/mL and, therefore, were moderately lower than the concentrations necessary to cause cytotoxic effects in eukaryotic cells. This suggests the possibility of a sufficient but narrow window for the potential application of these compounds.

All five newly synthesized alkyl pyridinol analogs are relatively lipophilic and are characterized by low molecular weights of 200–350 g/mol, resembling the characteristics of many membrane-permeable drugs [59]. We, therefore, predict that these compounds permeate the membrane and potentially interrupt intracellular metabolic processes, causing its bacteriostatic effects. JC-01-074 was particularly interesting to us as it was the only compound that exhibited bactericidal effects and, therefore, may differ in its mode of action. Further characterization of JC-01-074 revealed that it effectively causes significant damage and deformation to the staphylococcal membrane already at concentrations far below the MIC. These data suggest a mechanism of action for this compound that is associated with the bacterial membrane, which might be due to the cyclic amide of this compound. Cyclic amides, or lactams, are commonly utilized in drug discovery because of their versatility and use in a variety of potential therapeutic applications, such as diabetes, cancer, and infectious diseases [60]. The proximity of the nitrogen’s electron could allow it to be drawn into the secondary alcohol, which would increase the basicity and nucleophilicity and offer additional capabilities for hydrogen bond and salt formation. Incorporation of JC-01-074 into the cell membrane along with the formation of JC-01-074 salts or additional complexes could disrupt the osmotic equilibrium, impair the membrane potential, or mechanically perturb the membrane. Considering the findings from this study, along with the significance and relevance of pyridines in drug discovery, we believe that compounds such as JC-01-074 have the potential to serve as molecular templates for future efforts in drug development and discovery to treat staphylococcal infections more efficiently.

## 4. Materials and Methods

### 4.1. Strains and Growth Conditions

Strains used in this study are as follows: the *S. aureus* strain ATCC 25923, MRSA strains ATCC 33591, ATCC BAA-44, and USA300LAC, as well as the *P. aeruginosa* strain ATCC 27853. Unless indicated otherwise, tryptic soy broth (TSB) was inoculated with a single colony of the indicated strain and cultivated overnight at 37 °C and 300 rpm.

### 4.2. Synthesis of Alkyl Pyridinol Derivatives

The synthesis and characterization of the five alkyl pyridinol analogs are described in the Supplementary information (Supplementary Material and Methods, Supplementary Figures S4–S13).

### 4.3. Minimum Inhibitory Concentrations (MICs) of the Alkyl Pyridinol Derivatives in S. aureus and P. aeruginosa Strains

The MIC values of the alkyl pyridinol derivatives against the *S. aureus* strain ATCC 25923, MRSA strains ATCC 33591, ATCC BAA-44, and USA300LAC, as well as the *P. aeruginosa* strain ATCC 27853, were determined by following the CLSI guidelines for the broth microdilution antibiotic sensitivity testing protocol [30]. In brief, the overnight cultures were diluted into Mueller Hinton Broth (MHB) to a final inoculum concentration of 5 × 10^5^ CFU/mL (O.D_600nm_ = 0.00001) and dispensed into a 96-well polystyrene plate (Alkali Scientific, Fort Lauderdale, FL, USA). Alkyl pyridine compounds were diluted into inoculated MHB to the indicated concentrations from stock solutions that would allow for DMSO concentrations below 5%, resulting in a total volume of 200 μL per well. The plates were subsequently incubated at 37 °C for 16 h at 300 rpm. The MIC values, which are defined as the lowest compound concentration that inhibited growth, were determined from three biological replicates.

### 4.4. Minimum Bactericidal Concentrations (MBCs) of the Alkyl Pyridinol Derivatives in S. aureus

The MBC values for each alkyl pyridinol derivative were determined from the strains that had been cultivated with increasing concentrations of the respective compounds for 18 h. In brief, 10 µL of each culture was serially diluted and plated onto prewarmed LB agar and incubated overnight at 37 °C under static conditions to observe the colony-forming units (CFUs). The concentration at which no CFUs were observed was recorded as the MBC.

### 4.5. Growth Curve-Based Assay

The overnight USA300LAC cultures were diluted into fresh TSB media to an O.D._600nm_ = 0.001 and incubated at 37 °C under shaking conditions (300 rpm) until an O.D._600nm_ of 0.2–0.3 (early log phase) was reached. The cultures were subsequently transferred to a 96-well polystyrene plate (Alkali Scientific, Fort Lauderdale, FL, USA), following which DMSO (control) or alkyl pyridine compounds were added at the indicated final concentrations. The cells were then incubated at 37 °C for 16 h at 300 rpm, and the O.D._600nm_ measurements were taken every 10 min for 16 h in a Tecan Infinite 200 plate reader (Tecan, Morgan Hill, CA, USA).

### 4.6. Mammalian Cell Culture and MTT Assay

3T3 cells (male mouse embryonic fibroblast Flp-In cells, Thermo Fisher, Waltham, MA, USA) were cultured in D-MEM (Corning, Corning, NY, USA) supplemented with 10% Fetal Clone III (Cytiva Hyclone, Bengaluru, India) and L-Glutamine (Alfa Aesar, Haverhill, MA, USA) at 37 °C and 5% CO_2_. To determine the compound’s toxicity, 3T3 cells were seeded at a density of 10,000 cells/well into 96-well plates (Fisherbrand, Waltham, MA, USA) and cultured as described above. After 16 h, the culture medium was extracted and replaced by supplemented D-MEM without phenol red (Corning, Glendale, AZ, USA) containing individual compounds in the indicated concentrations. Furthermore, 1% sodium dodecyl sulfate (SDS) and 2% dimethyl sulfoxide (DMSO), equivalent to the volume of the DMSO in the highest compound concentration, were used as the cell death and vehicle control, respectively. Moreover, 16 h after compound treatment, the culture medium was extracted again and replaced by fresh supplemented D-MEM without phenol red. Subsequently, 10 µL of 5 mg/mL MTT reagent (Alfa Aesar, Haverhill, MA, USA), dissolved in DMSO, was added to each well, and the cells were incubated for 2 h at 37 °C and 5% CO_2_. Next, 85 µL of the culture supernatant was removed from each well, mixed with 50 µL of DMSO, and incubated in an optical grade 96-well plate (Fisherbrand, Waltham, MA, USA) in a shaking incubator at 37 °C and 250 rpm. The absorbance of the MTT-treated cell culture supernatant was measured at 540 nm in an ELx808 plate reader (Biotek, Winooski, VT, USA).

### 4.7. Propidium Iodide Fluorescence Assay

The plasma membrane integrity was determined using the Propidium Iodide Uptake assay, as previously described [61,62]. Briefly, the overnight cultures of USA300LAC were diluted into fresh TSB media, grown to an early log phase at 37 °C and 300 rpm, and treated with either DMSO, an alkyl pyridinol derivate, vancomycin, or SDS at the indicated concentrations for 1 h. Following two washing steps, the cells were then resuspended in phosphate-buffered saline (PBS), pH = 7.4, and subsequently stained with 1 µM propidium iodide (PI). The PI fluorescence intensity was determined at an excitation wavelength of 535 nm and an emission wavelength of 615 nm.

### 4.8. Fluorescence Microscopy

The overnight USA300LAC cultures were diluted into the TSB media to an O.D._600nm_ = 0.01 and incubated under shaking conditions at 37 °C until the mid-log phase (O.D._600nm_ = 0.3) was reached. The cultures were either left untreated or treated with DMSO, daptomycin (positive control), or the alkyl pyridinol derivative JC-01-074. The cells were collected at 15 and 60 min, respectively, washed twice, and resuspended in fresh PBS. The cells were subsequently stained with 5 µg/mL of DAPI and Nile Red for 10 min, washed twice, and resuspended in fresh PBS. The cells were then imaged on 1% agarose gel pads and via fluorescence microscopy using a Leica SP8 confocal microscope. A 63 ×/1.40 oil objective was used, with a white light laser set to a wavelength of 488 nm. Fluorescence was detected between the wavelengths of 500–560 nm, with an 8×- line averaging.

### 4.9. Inhibition of Biofilm Formation

The overnight USA300LAC cultures were diluted 1000-fold into the fresh TSB media and incubated under shaking conditions at 37 °C until the mid-log phase. The cultures were subsequently diluted to approximately 4 × 10^6^ CFU/mL and dispended into 96-well polystyrene plates (Alkali Scientific, Fort Lauderdale, FL, USA) containing DMSO (control), vancomycin (0.5–4× MIC), or the indicated alkyl pyridinol compounds (0.5–4× MIC) and sealed with a breath-easy membrane (Fisherbrand, Waltham, MA, USA). The peripheral wells were filled with PBS to limit evaporation. The plates were incubated without agitation at 37 °C for 24 h, following which the planktonic cells were removed. The biofilms were washed twice with PBS, stained with 0.1% crystal violet (CV) for 15 min, and washed again twice with PBS to remove the unbound CV dye. CV bound to biofilm cells was extracted with 30% (*v*/*v*) glacial acetic acid, and the O.D._595nm_ was determined.

### 4.10. Biofilm Eradication Assay

The overnight USA300LAC cultures were diluted 1000-fold into the TSB media and incubated under shaking conditions at 37 °C until the mid-log phase. The cultures were subsequently diluted to approximately 4 × 10^6^ CFU/mL and dispended into 96-well polystyrene plates (Alkali Scientific, Fort Lauderdale, FL, USA). The peripheral wells were filled with PBS to limit evaporation. The plates were sealed with a breath-easy membrane (Fisher Scientific) and incubated without agitation at 37 °C for 24 h, following which the planktonic cells were removed and the biofilms washed twice with PBS. Subsequently, the biofilms were incubated under static conditions with the TSB media, either containing DMSO (control), vancomycin (0.5–4× MIC), or one of the indicated alkyl pyridinol compounds (0.5–4× MIC). After 24 h of incubation, biofilm eradication was assessed via CV staining at 595 nm in a Tecan 200 Infinite Pro microplate reader (Tecan, Morgan Hill, CA, USA).

### 4.11. Evaluation of Antibiotic Synergy with JC-01-074

Following previous studies, the checkerboard assay [48] was employed to determine whether JC-01-074 had synergistic effects in combination with members of different antibiotic classes. The indicated antibiotics tested in combination were first serially diluted two-fold in MHB on a longitudinal axis in a 96-well polystyrene plate. JC-01-074 was then serially diluted two-fold across the 96-well plate horizontal axis. The plates were inoculated with USA300LAC to a final inoculum concentration of 5 x10^5^ CFU/mL (O.D._600nm_ = 0.0001) and incubated overnight at 37 °C for 20 h. The growth was measured at 600 nm in a Tecan 200 Infinite Pro microplate reader. The fractional inhibitory concentration was calculated as follows:

FIC = MIC_JC-01-074 + antibiotics_/MIC_JC-01-074_

The classification of interactions was based on the FIC index: interactions were categorized as synergistic (FIC ≤ 0.5), additive/no interaction (FIC 0.5–2.0), and antagonistic (FIC > 2.0).

## Figures and Tables

**Table 1:** Minimal inhibitory concentrations (MIC) and minimal bactericidal concentrations (MBC) of the indicated compounds tested against Gram-negative *P. aeruginosa* and Gram-positive *S. aureus*/MRSA strains. MIC and MBC values were determined as described in the Material and Methods. ND, not determined.

Compound	*P. aeruginosa* ATCC 27853	*S. aureus* ATCC 25923	*MRSA* ATCC 33591	*MRSA* ATCC BAA-44	*MRSA* USA300LAC	*MRSA* USA300LAC
	MIC (µg/mL)	MIC (µg/mL)	MIC (µg/mL)	MIC (µg/mL)	MIC (µg/mL)	MBC (µg/mL)
**JC-01-072**	>128	4	4	4	8	>128
**EA-02-009**	>128	1	0.5	1	32	>128
**JC-01-074**	>128	16	16	16	16	16
**JC-01-083**	>128	>128	>128	>128	ND	ND
**EA-02-011**	>128	32	32	32	ND	ND
**Vancomycin**	ND	ND	ND	ND	1	ND
**Ampicillin**	ND	ND	ND	ND	2.33	ND
**Chloramphenicol**	ND	ND	ND	ND	4	ND
**Gentamycin**	ND	ND	ND	ND	1	ND
**Ciprofloxacin**	ND	ND	ND	ND	16	ND
**Doxycycline**	ND	ND	ND	ND	0.125	ND

**Table 2:** Effect of alkyl pyridine compounds on the viability of 3T3 cells upon treatment for 24 h. 3T3 cells were incubated with different concentrations of the indicated compounds for 24 h before cell viability was assessed using the MTT assay. Treatment with 1% SDS and 2% DMSO served as cell death and vehicle control, respectively.

Compound	IC50 [mM]	[µg/mL]	*n*
**JC-01-072**	153.2 ± 33.8	37.7 ± 8.3	4
**EA-02-009**	106.6 ± 82.3	34.5 ± 26.7	4
**JC-01-074**	103.9 ± 9.4	25.6 ± 2.3	3
**JC-01-083**	78.2 ± 24.0	31 ± 9.5	4
**EA-02-011**	43.6 ± 8.3	10.7 ± 2	3

## Data Availability

The original contributions presented in the study are included in the article/Supplementary Material, further inquiries can be directed to the corresponding author.

## Supplementary Materials

The following supporting information can be downloaded at: https://www.mdpi.com/article/10.3390/antibiotics13090897/s1.
