# Supplementary material for: Alkyl Pyridinol Compounds Exhibit Antimicrobial Effects against Gram-Positive Bacteria
Source: Antibiotics (Basel). 2024 Sep 20;13(9):897. doi: 10.3390/antibiotics13090897 (PMC11428593; doi:10.3390/antibiotics13090897)
Supplement: Supplementary file 1 [file antibiotics-13-00897-s001.zip › antibiotics-3210148-supplementary.pdf]

SUPPLEMENTARY INFORMATION

MATERIALS AND METHODS

General experimental procedures for synthesis and characterization of the analogues. ^{1}H and ^{13}C NMR spectra were obtained on a Bruker 400 MHz Avance III spectrometer or Bruker 500 MHz Avance III spectrometer in $CDCl_3$ unless otherwise noted. Chemical shifts are reported with the residual solvent peak used as an internal standard ($CDCl_3$ = 7.26 ppm for ^{1}H and 77.16 ppm for ^{13}C). High-resolution mass spectra were obtained using positive mode electrospray ionization (ESI+) on a Thermo Scientific Q Exactive hybrid Quadripole-Orbitrap mass spectrometer. Reactions were monitored by TLC analysis (silica gel 60 F254, 250 mm layer thickness) and visualized with a 254 nm UV light. Flash column chromatography was performed with silica gel 60 (230-400 mesh). All starting materials and solvents were purchased from commercial chemical companies and used as instructed by the vendor.

Synthesis of 5-(undec-1-yn-1-yl) pyridin-3-ol (**JC-01-072**). To a solution of 5-iodopyridine-3-ol (100 mg, 0.45 mmol) in acetonitrile (2.15 mL) was added 1-undecyne (0.27 mL, 1.36 mmol), solid CuI (17.2 mg, 0.09 mmol) and $PdCl_2(PPh_3)_2$ (15.9 mg, 0.023 mmol). Then, Et_3N (0.21 mL, 1.50 mmol) was added dropwise, and the reaction was left to stir at room temperature for 1 hr and at 60 °C for 3 hrs. A mixture of product and Triethylamine salt resulted, was quenched with saturated $NaHCO_3$ (10 mL) and the aqueous layer was extracted with Ethyl acetate (3 × 10 mL). The combined organic layers were washed with brine, dried ($MgSO_4$), filtered, and concentrated under reduced pressure. The residue was purified by flash column chromatography (0-100% Ethyl acetate /Hexanes) to yield **JC-01-072** as a brown-yellow solid **(**53.0 mg, 47 % yield): ^{1}H NMR ($CDCl_3$, 400 MHz) δ_H 8.11 (s, 2H), 7.28 (s, 1H), 2.40 (t, *J* = 7.1 Hz, 2H), 1.60 (p, *J* = 6.9 Hz, 2H), 1.43 (p, *J* = 6.8 Hz, 2H), 1.34-1.28 (m, 10H); 0.88 (t, *J* = 6.7 Hz, 3H); ^{13}C NMR ($CDCl_3$, 100 MHz) δ_C 154.4, 142.4, 135.2, 127.3, 123.0, 94.9, 77.4, 32.0, 30.0, 29.4, 29.3, 29.1, 28.7, 22.8, 19.6, 14.2; HRESIMS *m*/*z* 246.1857 $[M+H]^+$ (calcd for $C_{16}H_{23}NO$, 246.1858).

Synthesis of 6-(undec-1-yn-1-yl) pyridin-2-ol (**JC-01-074).** To a solution of 6-iodopyridine-2-one, (100 mg, 0.45 mmol) in acetonitrile (2.15 mL) was added 1-undecyne (0.27 mL, 1.36 mmol), solid CuI (17.2 mg, 0.09 mmol) and $PdCl_2(PPh_3)_2$ (15.9 mg, 0.023 mmol). Then mL Et_3N (0.21 mL, 1.50 mmol) was added dropwise, and the reaction was left to stir at room temperature for 1 hr and at 60 °C for 3 hrs. A mixture of product and Triethylamine salt resulted, was quenched with saturated $NaHCO_3$ (10 mL) and the aqueous layer was extracted with Ethyl acetate (3 × 10 mL). The combined organic layers were washed with brine, dried ($MgSO_4$), filtered, and concentrated under reduced pressure. The residue was purified by flash column

chromatography (0-100% Ethyl acetate /Hexanes) to yield **JC-01-074** as a yellow solid (10.2 mg, 9.2% yield) :^{1}H NMR ($CDCl_3$, 400 MHz) δ_H 7.29 (d, *J* = 6.5 Hz, 1H), 6.58 (s, 1H), 6.24 (d, *J* = 6.4 Hz, 1H), 2.41 (t, *J* = 7.1 Hz, 2H), 1.60 (p, *J* = 7 Hz, 2H), 1.42 (p, *J* = 6.8 Hz, 2H), 1.34-1.25 (m, 10H), 0.86 (t, *J* = 6.6 Hz, 3H); ^{13}C NMR ($CDCl_3$, 100 MHz) δ_C 164.9, 138.4, 134.2, 122.5, 110.3, 98.3, 78.4, 32.0, 30.0, 29.4, 29.2, 29.1, 28.4, 22.8, 19.7, 14.2; HRESIMS *m/z* 246.1854 [M+H]$^+$ (calcd for $C_{16}H_{23}NO$, 246.1858).

Synthesis of 2-bromo-6-(undec-1-yn-1-yl) pyridin-4-ol (**EA-02-009**). To a solution of 2,6-dibromopyridine-ol (75.0 mg, 0.29 mmol) in acetonitrile (2.15 mL) was added 1-undecyne (0.16 mL, 0.89 mmol, solid CuI (11.3 mg, 0.059 mmol) and $PdCl_2(PPh_3)_2$ (10.4 mg, 0.015 mmol). Then Et_3N (0.14 mL, 0.98 mmol) was added dropwise, and the reaction was left to stir at room temperature for 1 hr and at 60 °C for 3 hrs. A mixture of product and Triethylamine salt resulted, was quenched with $NaHCO_3$ (10 mL), and the aqueous layer was extracted with Ethyl acetate (3 × 10 mL). The combined organic layers were washed with brine, dried ($MgSO_4$), filtered, and concentrated under reduced pressure. The residue was purified by flash column chromatography (0-100% Ethyl acetate /Hexanes) to yield **EA-02-009** as a brown oil (24.1 mg, 32 % yield): ^{1}H NMR ($CDCl_3$, 400 MHz) δ_H 7.01 (d, *J* = 1.5 Hz, 1H), 6.90 (d, *J* = 1.6 Hz, 1H), 2.34 (t, *J* = 7.1 Hz, 2H), 1.52 (p, *J* = 7.0 Hz, 2H), 1.37-1.26 (m, 12 H), 0.88 (t, *J* = 6.2 Hz, 3H); ^{13}C NMR ($CDCl_3$, 125 MHz) δ_C 175.1, 139.7, 133.2, 115.7, 115.3, 95.0, 77.5, 31.9, 29.4, 29.3, 29.1, 28.9, 28.1, 22.7, 19.3, 14.1; HRESIMS *m/z* 324.0958 [M+H]$^+$ (calcd for $C_{16}H_{22}BrNO$, 324.0963).

Synthesis of 2,6-di(undec-1-yn-1-yl) pyridin-4-ol (**JC-01-083).** To a solution of 2,6-dibromopyridine-ol (75.0 mg, 0.29 mmol) in acetonitrile (2.15 mL) was added 1-undecyne (0.16 mL, 0.89 mmol, solid CuI (11.3 mg, 0.059 mmol) and $PdCl_2(PPh_3)_2$ (10.4 mg, 0.015 mmol). Then Et_3N (0.14 mL, 0.98 mmol) was added dropwise, and the reaction was left to stir at room temperature for 1 hr and at 60 °C for 3 hrs. A mixture of product and Triethylamine salt resulted, was quenched with $NaHCO_3$ (10 mL), and the aqueous layer was extracted with Ethyl acetate (3 × 10 mL). The combined organic layers were washed with brine, dried ($MgSO_4$), filtered, and concentrated under reduced pressure. The residue was purified by flash column chromatography (0-100% Ethyl acetate/Hexanes) to yield **JC-01-083** as a brown-yellow oil (16.0 mg, 21 % yield): ^{1}H NMR ($CDCl_3$, 400 MHz) δ_H 6.67 (s, 2H), 2.35 (t, *J* = 7.1 Hz, 4H), 1.54 (p, *J* = 7.1 Hz, 4H), 1.39-1.26 (m, 24H), 0.88 (t, *J* = 6.6 Hz, 6H); ^{13}C NMR ($CDCl_3$, 100 MHz) δ_C 161.1, 137.0, 118.4, 96.7, 77.4, 32.0, 30.0, 29.4, 29.3, 29.1, 28.2, 22.8, 19.5, 14.2; HRESIMS *m/z* 396.3264 [M+H]$^+$ (calcd for $C_{27}H_{41}NO$, 396.3266).

Synthesis of 2-(undec-1-yn-1-yl) pyridin-4-ol (**EA-02-011**). To a solution of 2-bromo-6-(undec-1-yn-1-yl) pyridin-4-ol (**EA-02-009**) (42.8 mg, 0.13 mmol) in dry THF (0.70 mL) at –78 °C, was added n-BuLi (0.12 mL, 1.32 mmol) dropwise. The reaction was left to stir for 20 minutes, then quenched with saturated NH_4Cl

(10mL) and extracted with Ethyl ether (3 × 10 mL). The combined organic layers were washed with brine, dried ($MgSO_4$) and concentrated. The reaction was purified under silica gel column chromatography using (0-10 % Methanol/Dichloromethane) to yield **EA-02-011** as a brown oil (12.4 mg, 38% yield): ^{1}H NMR ($CDCl_3$, 400 MHz) δ_H 7.67 (d, J = 6.9 Hz. 1H), 6.65 (d, J = 2.2 Hz, 1H), 6.51 (dd, J = 6.9, 2.3 Hz, 1H), 2.33 (t, J = 7.1 Hz, 2H), 1.52 (p, J = 7.0 Hz, 2H), 1.37-1.24 (m, 12H), 0.86 (t, J = 6.6 Hz, 3H); ^{13}C NMR ($CDCl_3$, 100 MHz) δ_C 177.4, 140.6, 135.7, 119.9, 115.8, 96.3, 75.2, 32.0, 29.6, 29.4, 29.3, 29.1, 28.2, 22.8, 19.5, 14.2; HRESIMS *m*/*z* 246.1853 $[M+H]^+$ (calcd for $C_{16}H_{23}NO$, 246.1858).

SUPPLEMENTARY FIGURES

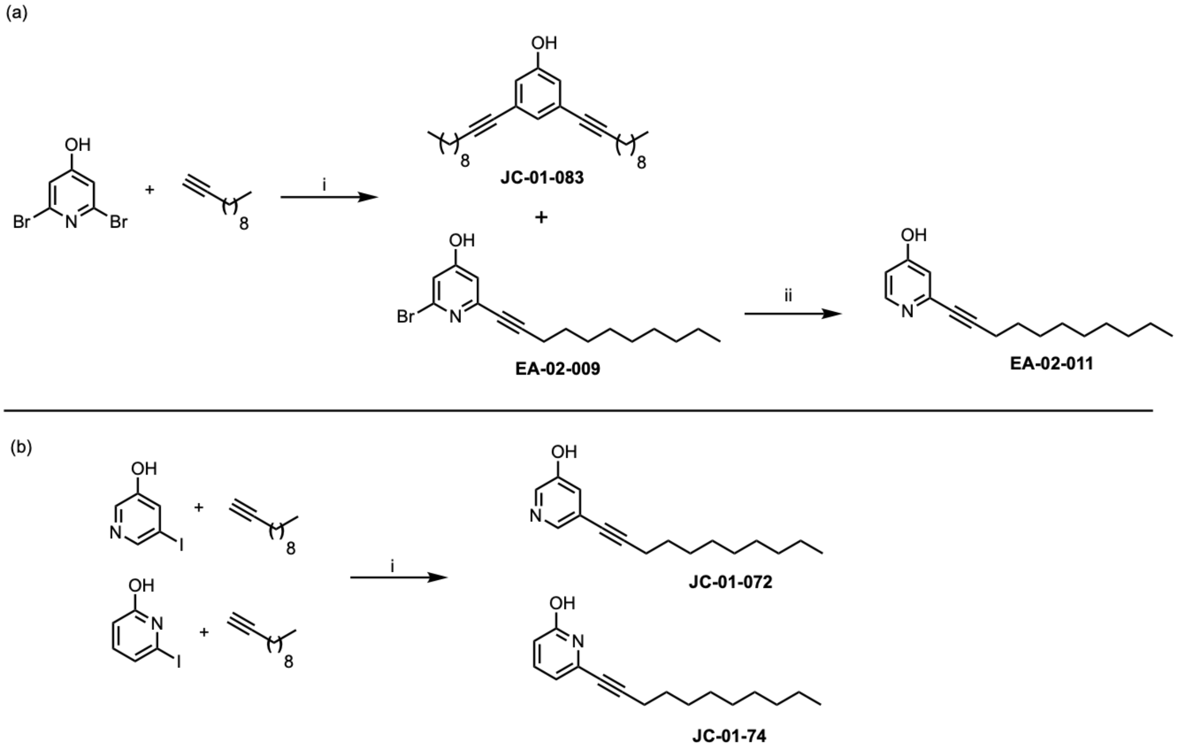

Figure S1. **Synthetic route used to generate anaephene analogues. (a)** Analogues **JC-01-083 & EA-02-009** were generated in a single reaction (step i), while synthesis of **EA-02-011** required a subsequent step (step ii). **(b)** Single step synthesis used to generate analogues **JC-01-072** & **JC-01-074.** Reagents and conditions: (i) CuI, Et_3N, $PdCl_2(PPh_3)_2$, MeCN, 24 hr, rt; (ii): n-BuLi, THF, −78°C for 30 min.

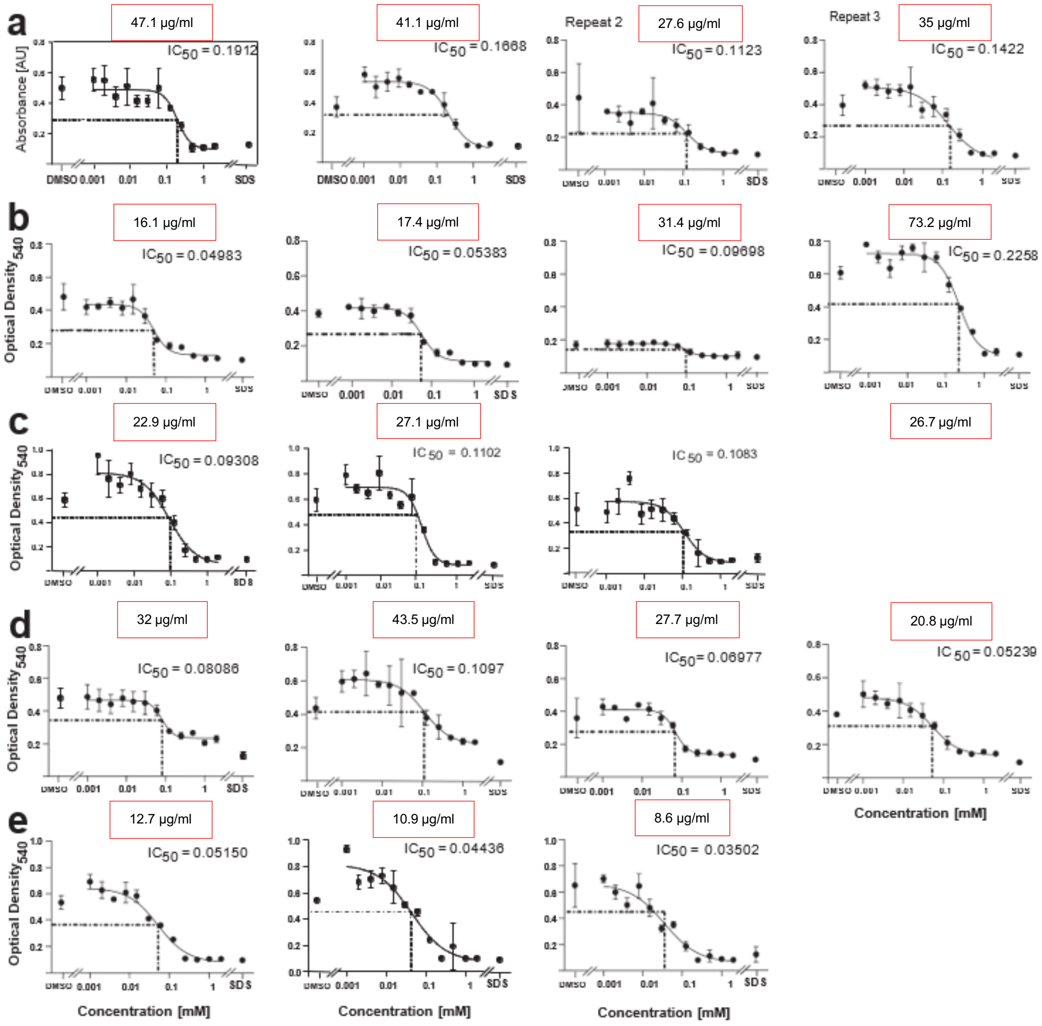

Figure S2. Cytotoxicity of the alkyl pyridinol compounds tested on 3T3 cells with the MTT assay. 3T3 cells were treated with increasing concentrations of the indicated alkyl pyridinol compound for 24 hours before cell viability was assessed with the MTT assay. Cells treated with 1% SDS and 2% DMSO were used as positive and vehicle controls, respectively. Graphs show the mean $A_{540\ nm}$ of supernatant from cells treated with JC-01-72 (**a**), EA-02-09 (**b**), JC-01-74 (**c**), JC-01-83 (**d**), and EA-02-11 (**e**). Averages are derived from 3-4 biological replicates, each containing 3 technical replicates: *(n = 3-4, ± S.D.)*. IC_{50} values are given in mM.

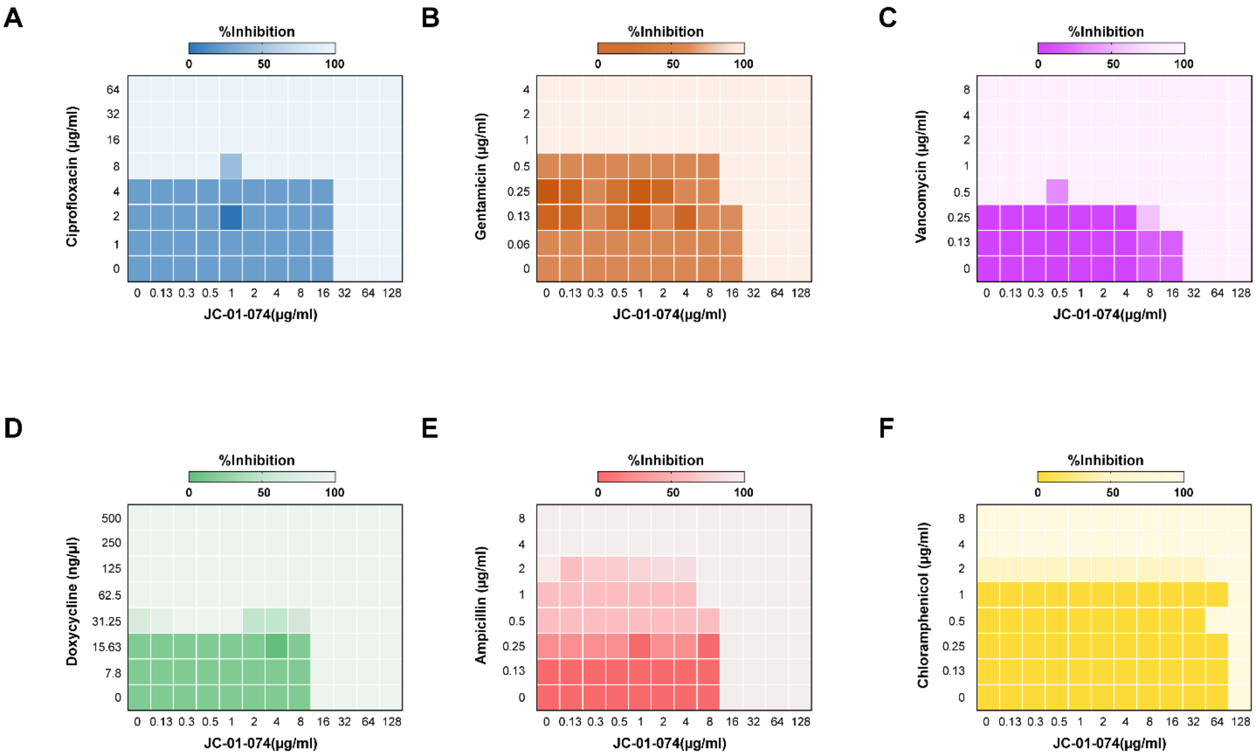

Figure S3. Checkerboard assays with JC-01-074. JC-01-074 was tested against *S. aureus* strain USA300LAC in combination with ampicillin **(A)**, gentamicin **(B)**, ciprofloxacin **(C)**, doxycycline **(D)**, vancomycin **(E)**, and chloramphenicol **(F)** to determine possible synergistic effects when combined with conventional antibiotics. Antibiotics tested in combination were initially serially diluted two-fold in MHB on a longitudinal axis in a polystyrene 96 well plate. JC-01-074 was then serially diluted two-fold across the 96 well plate horizontal axis before cells were cultivated at 37 ˚C and 200 rpm for 20 hrs and optical densities at 600 nm determined in a Tecan plate reader. FICs were calculated as the ratio of the MIC of the antimicrobial in combination over the MIC of the antimicrobial alone. Interactions were categorized as synergistic (<0.5), additive or no interaction (0.5 - 2), and antagonistic (>2).

FIGURES S4-S13:

^{1}H NMR spectrum of Compound **EA-02-009**…………………………………………… S4

^{13}C NMR spectrum of Compound **EA-02-009**……………………………………………S5

^{1}H NMR spectrum of Compound **EA-02-011**…………………………………………… S6

^{13}C NMR spectrum of Compound **EA-02-011**……………………………………………S7

^{1}H NMR spectrum of Compound **JC-01-072**……………………………………………. S8

^{13}C NMR spectrum of Compound **JC-01-072**…………………………………………… S9

^{1}H NMR spectrum of Compound **JC-01-074**……………………………………………. S10

^{13}C NMR spectrum of Compound **JC-01-074**……………………………………………. S11

^{1}H NMR spectrum of Compound **JC-01-083**…………………………………………… S12

^{13}C NMR spectrum of Compound **JC-01-083**……………………………………………S13

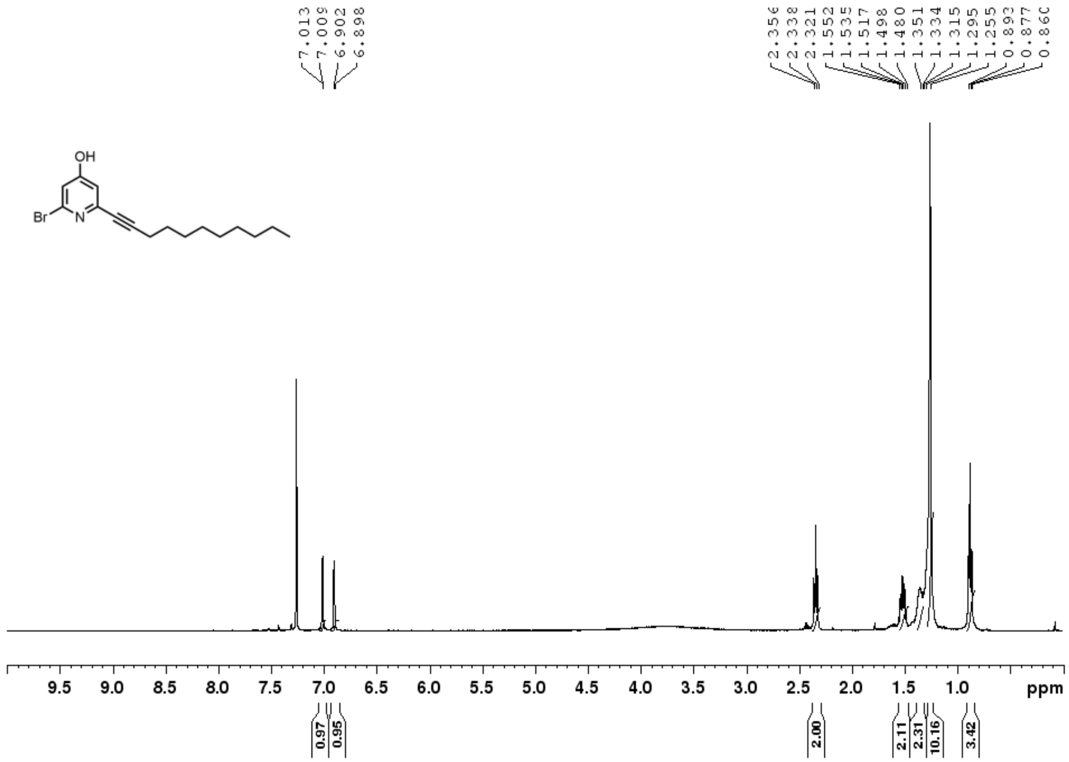

Figure S4. ^{1}H NMR spectrum of **EA-02-009** in $CDCl_3$.

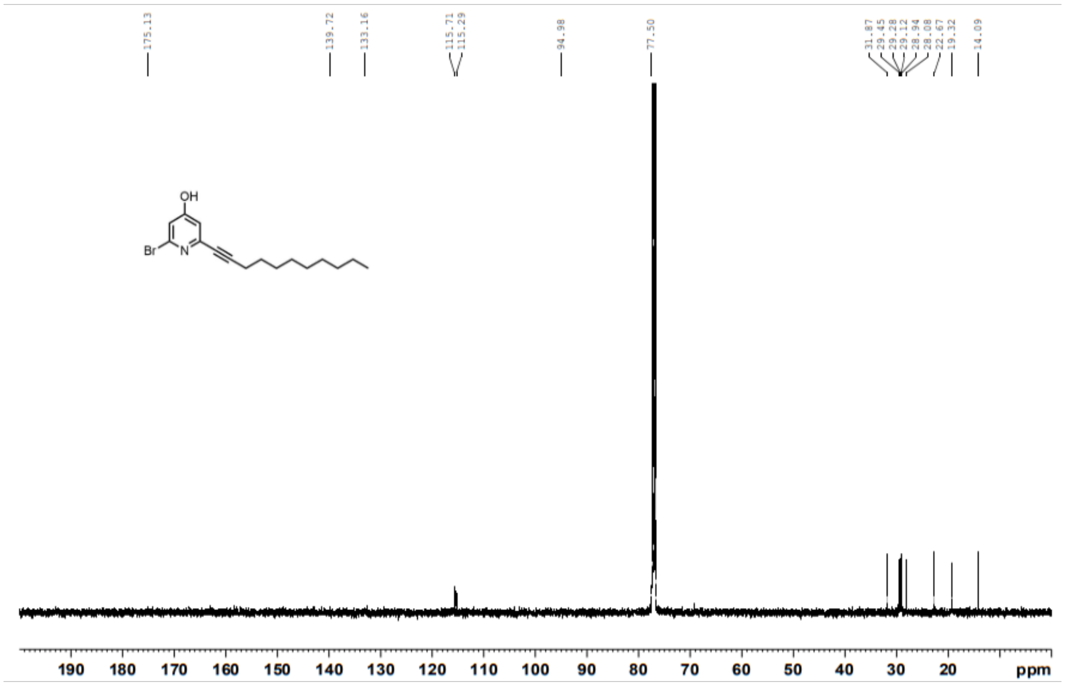

Figure S5. ^{13}C NMR spectrum of **EA-02-009** in $CDCl_3$.

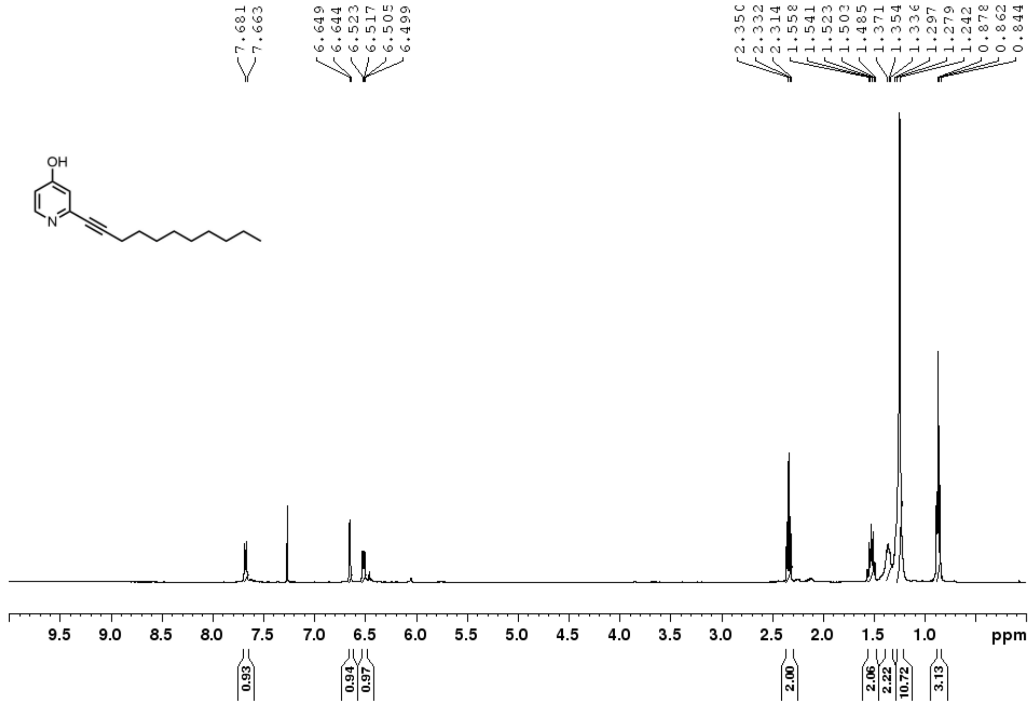

Figure S6. ^{1}H NMR spectrum of **EA-02-011** in $CDCl_3$.

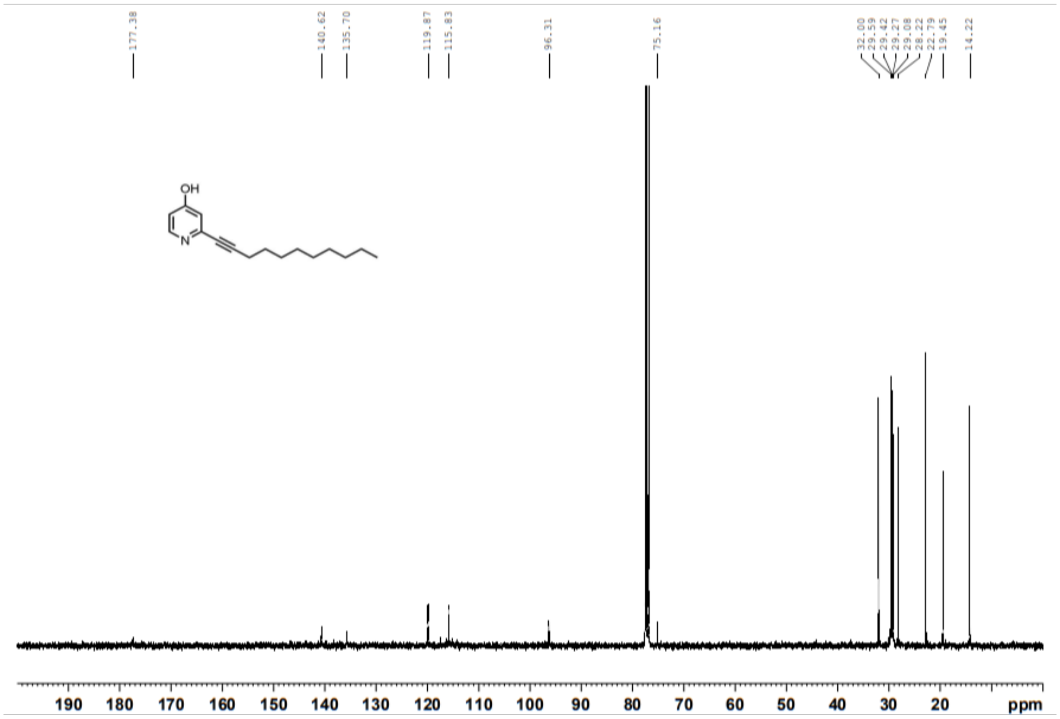

Figure S7. ^{13}C NMR spectrum of **EA-02-011** in $CDCl_3$.

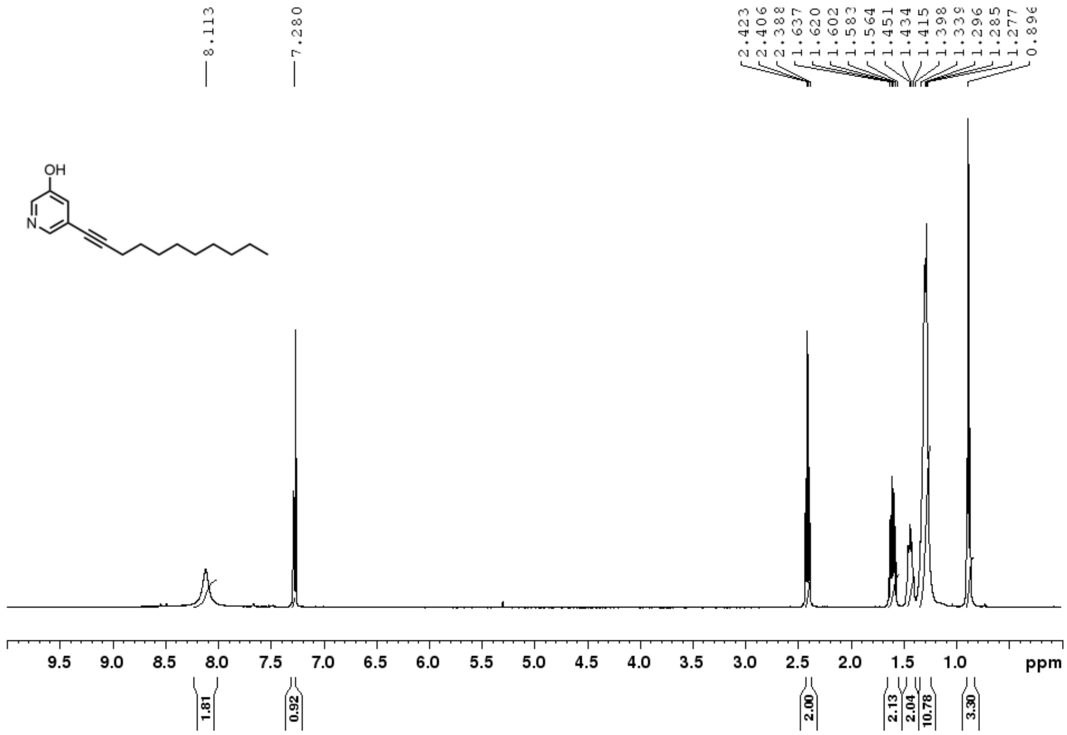

Figure S8. ^{1}H NMR spectrum of **JC-01-072** in $CDCl_3$.

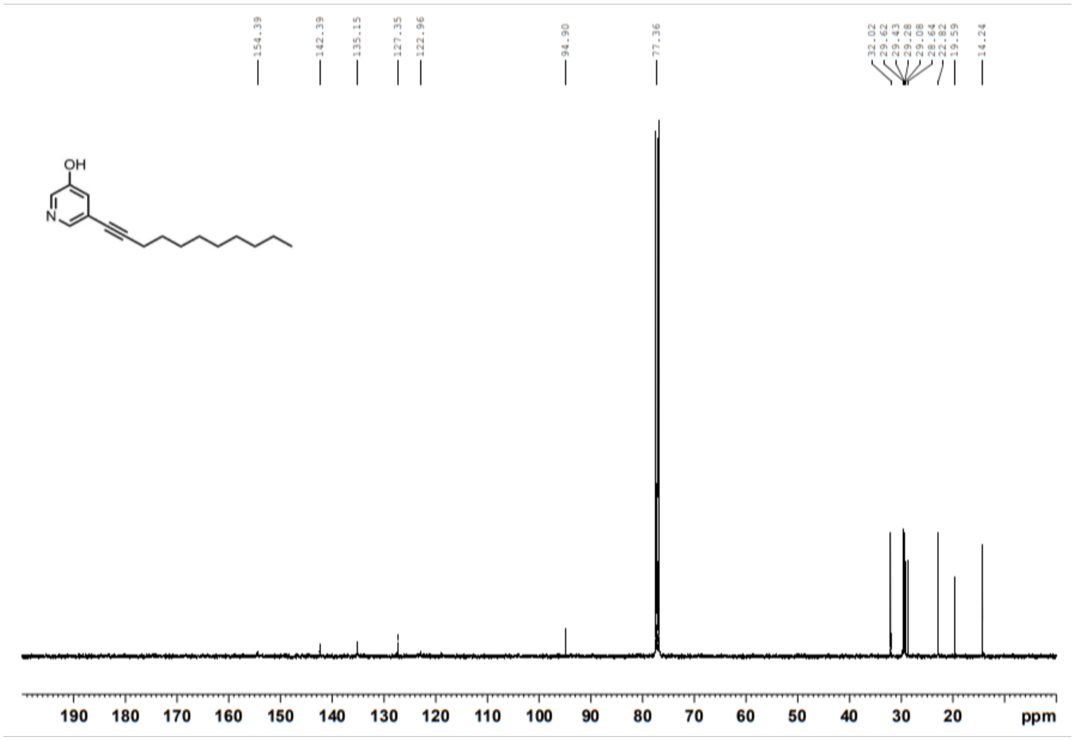

Figure S9. ^{13}C NMR spectrum of **JC-01-072** in $CDCl_3$.

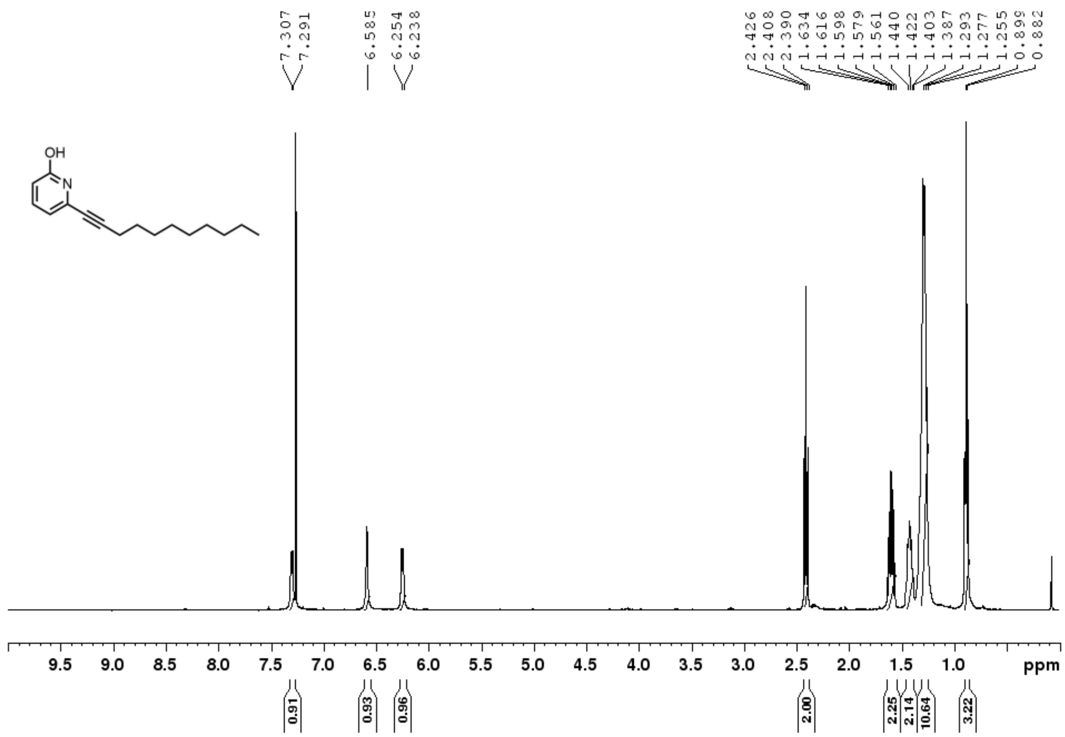

Figure S10. ^{1}H NMR spectrum of **JC-01-074** in $CDCl_3$.

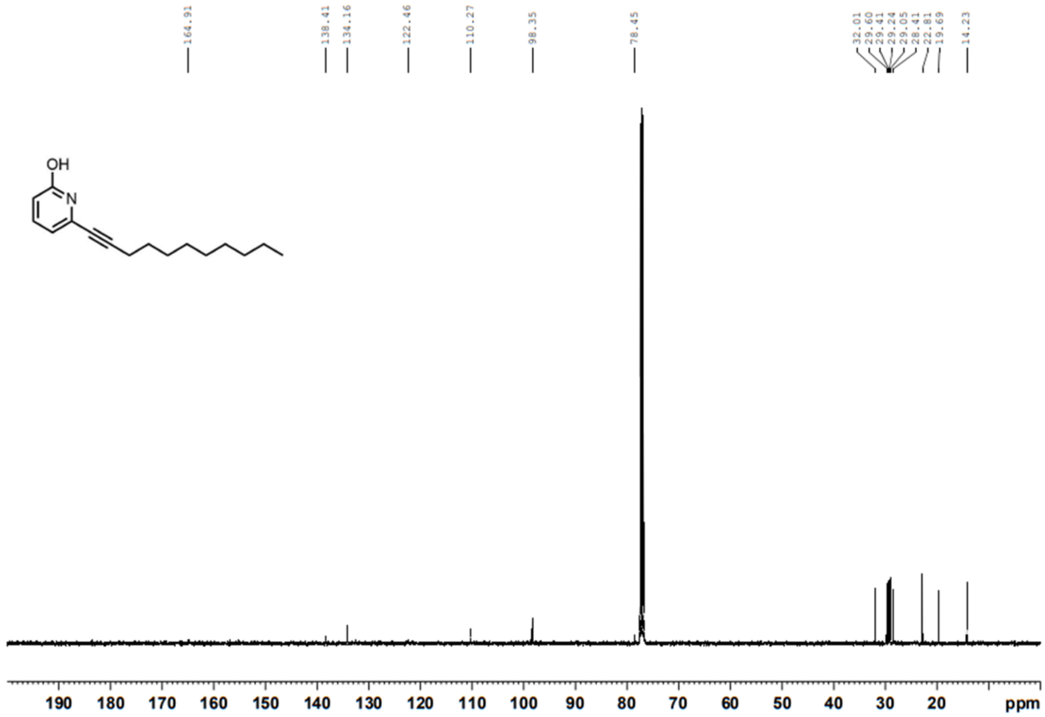

Figure S11. ^{13}C NMR spectrum of **JC-01-074** in $CDCl_3$.

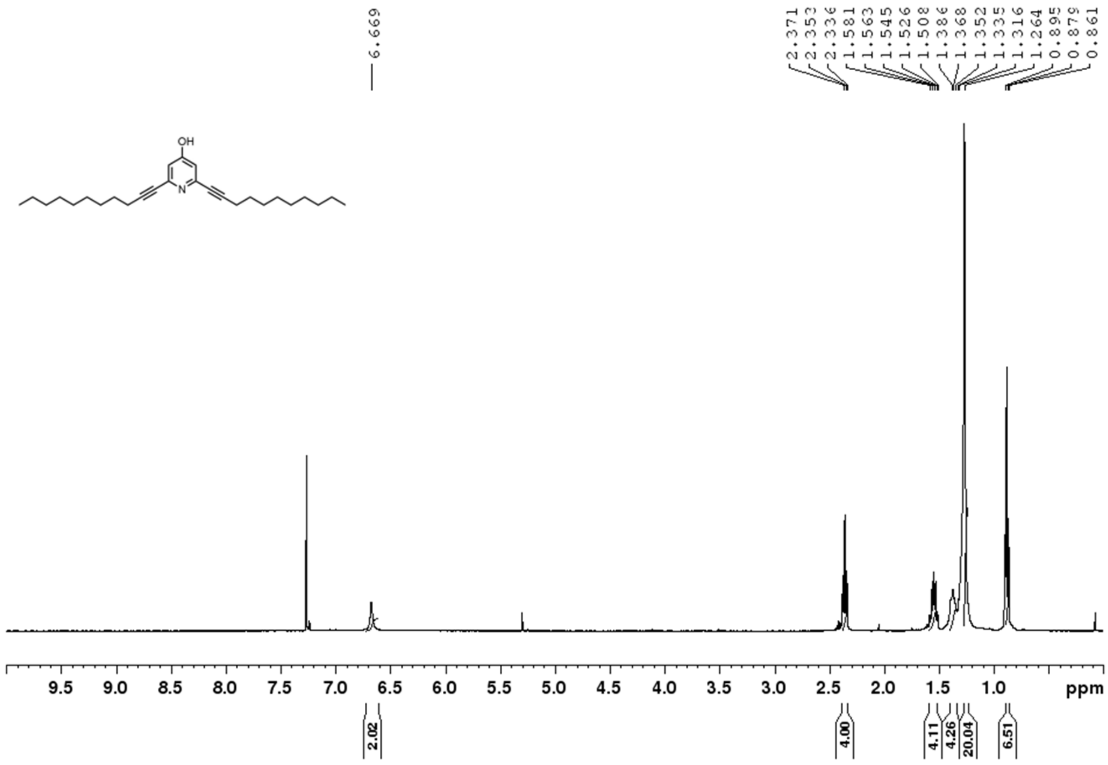

Figure S12. ^{1}H NMR spectrum of **JC-01-083** in $CDCl_3$.

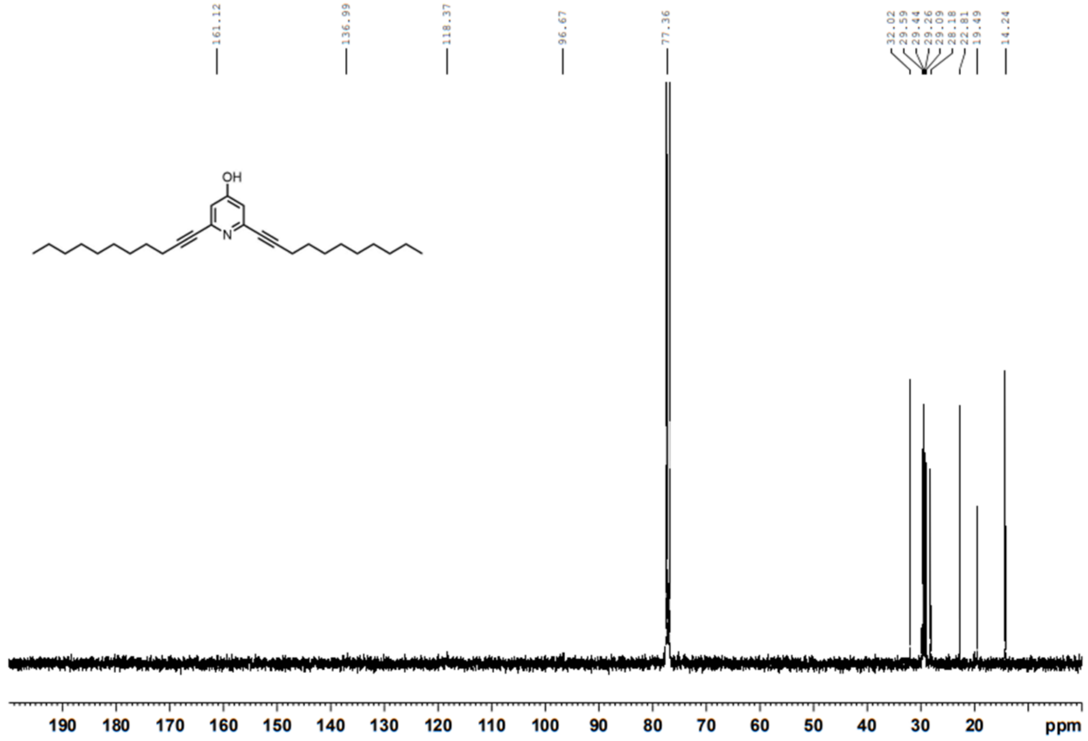

Figure S13. ^{13}C NMR spectrum of **JC-01-083** in $CDCl_3$.
